# Supplementary material for: Trajectories of change after a health-education program in Japan: decay of impact in anxiety, depression, and patient-physician communication
Source: PeerJ. 2019 Jul 15;7:e7229. doi: 10.7717/peerj.7229 (PMC6637923; doi:10.7717/peerj.7229)
Supplement: Supplemental Information 1 — In Appendix 1, Table A1A shows basic descriptive statistics regarding the participants’ history of illness, age, and number of diagnoses; Figure A1A shows the frequency distribution of the history of illness; Figure A1B shows the frequency distribution of age; Figure A1C shows the frequency distribution of the number of diagnoses; Table A1B shows the frequencies of the numbers of diagnoses; and Table A1C lists the diagnoses and the number of participants with each diagnosis. Appendix 2 gives more details of the results of the growth-mixture modeling (GMM). In Appendix 2, Tables A2A–A2C show the Bayesian information criterion (BIC) the classification index (Pc), and the equations for each of four models of the anxiety data, the depression data, and the communication data, respectively. Appendix 3 shows contingency tables and Phi coefficients for trajectory-group memberships (Table A3A, anxiety-depression; Table A3B, anxiety-communication; Table A3C, depression-communication). [file peerj-07-7229-s001.pdf]

Appendix 1.

Table A1A. Participants in the study: basic descriptive statistics

Statistic	History of illness (years)	Age (years)	Number of Diagnoses
**Valid n**	441	453	456
**Number Missing**	15	3	0
**Mean**	13.64	48.79	1.774
**Median**	10.00	48.00	1.000
**Std. Deviation**	12.15	14.03	1.052
**Skewness**	1.313	0.05549	1.633
**Std. Error of Skewness**	0.1162	0.1147	0.1143
**Kurtosis**	1.559	-0.7745	2.972
**Std. Error of Kurtosis**	0.2320	0.2289	0.2282
**Minimum**	0.000	18.00	1.000
**25th percentile**	4.000	38.00	1.000
**50th percentile**	10.00	48.00	1.000
**75th percentile**	20.00	59.00	2.000
**Maximum**	63.00	83.00	7.000

Number (%) of women: 362 (79%)

Number (%) living with spouse or partner: 238 (52%)

Figure A1A. Frequency distribution of history of illness (years)

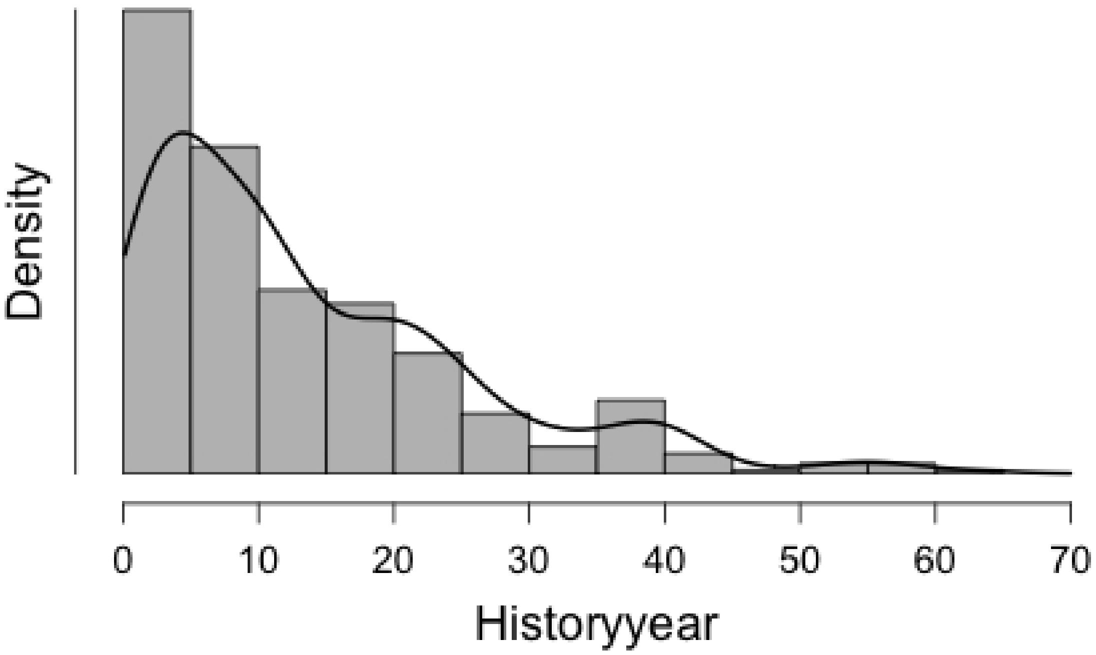

Figure A1B. Frequency distribution of age (years)

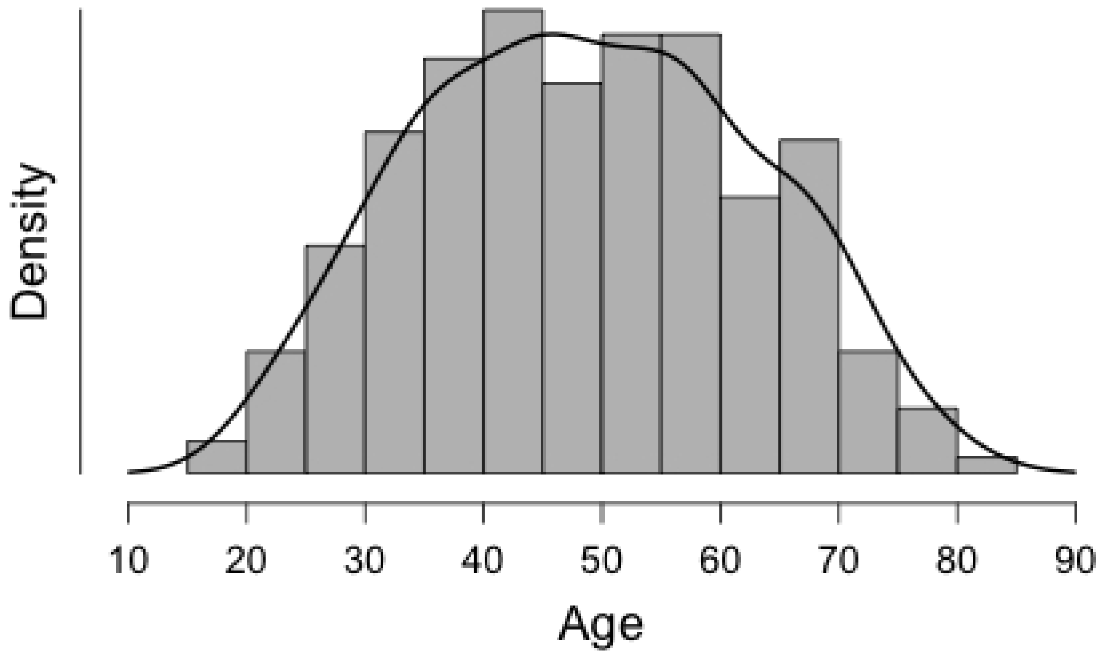

Figure A1C. Frequency distribution of number of diagnoses

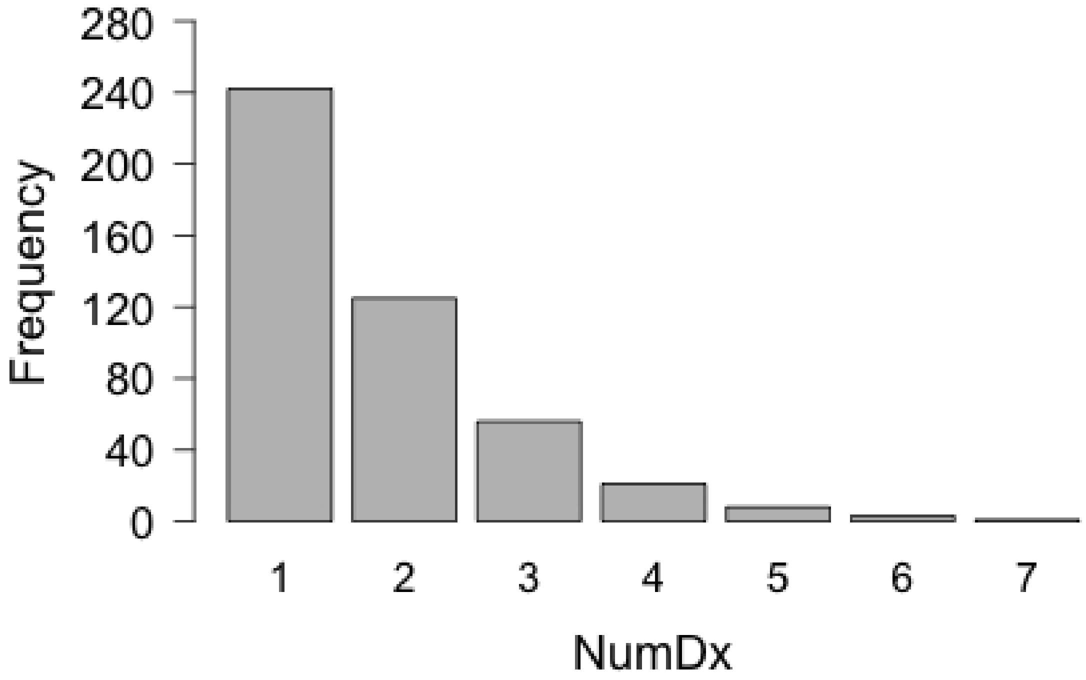

Table A1B. Frequencies of numbers of diagnoses

Number of Diagnoses	Frequency	Percent	Cumulative Percent
1	242	53.1	53.1
2	125	27.4	80.5
3	56	12.3	92.8
4	21	4.6	97.4
5	8	1.8	99.1
6	3	0.7	99.8
7	1	0.2	100.0
Total	456	100.0	

Table A1C. Diagnoses, ordered by the number of participants with each diagnosis

Diagnosis	Number of participants
Allergic disease	120
Cardiovascular disease	96
Connective tissue disease	85
Diabetes mellitus	83
Rheumatoid arthritis	51
Fibromyalgia syndrome	38
Cancer	26
Depression	26
Asthma	23
Inflammatory bowel disease	21
Pulmonary disease	21
Parkinson’s disease	13

Appendix 2.

In this study the decision about the number of groups was based on the BIC and the P_c, but in some circumstances practical considerations could override the conclusions from those statistical criteria. For example, if only very limited resources are available to give reinforcement to the participants who are at risk of decay of impact, then a three-group model might be preferred. The reason is that the three-group model identified a group that begins from a much worse baseline value and is also more "volatile," that is, it has both greater improvement and greater decay. That group is smaller – about one third rather than one half of the total. The limited resources could then be focused on that relatively small group with the greatest need. Because there might be such a practical reason for using three-group results, and for the readers' general information, those results are given in the Tables here in Appendix 2.
This Appendix shows the results of Growth-Mixture Modeling.
1. BIC is the Bayesian Information Criterion, estimated using the deviance (–2 log likelihood). The model with the lowest BIC is preferred.
2. Classification (P_c) is the proportion correctly classified, based on posterior probability. The model with the highest P_c is preferred [Clogg, C. C. (1995). Latent class models. In G. Arminger, C. C. Clogg, & M. E. Sobel (Eds.), Handbook of statistical modeling for the social and behavioral sciences (pp. 311-359). New York: Plenum Press].
3. Within each model that has more than one group, the equations are listed in order of increasing baseline value (Y intercept).

Table A2A.
Four models of the Anxiety data

Number of groups in the model	Model fit statistics BIC	Classification (P_c)	Equation(s) for the group(s) in the model
1	9,284.47	Not applicable	$Y = 6.8872 - 0.244*X + 0.0181*X^2$
2	9,186.65	.88	$Y = 4.3745 - 0.0888*X + 0.0028*X^2$<br>$Y = 9.6698 - 0.4115*X + 0.0349*X^2$
3	9,218.07	.85	$Y = 3.1803 - 0.0344*X - 0.0014*X^2$<br>$Y = 6.8929 - 0.1462*X + 0.0064*X^2$<br>$Y = 10.6924 - 0.5619*X + 0.0506*X^2$
4	9,273.20	.69	$Y = 2.9112 - 0.0467*X - 0.0007*X^2$<br>$Y = 6.4363 - 0.3146*X + 0.018*X^2$<br>$Y = 8.6719 - 0.0115*X + 0.0034*X^2$<br>$Y = 9.8725 - 0.602*X + 0.0523*X^2$

Table A2B.
Four models of the Depression data

Number of groups in the model	Model fit statistics		Equation(s) for the group(s) in the model
	BIC	Classification (P_c)	
1	9,022.85	Not applicable	$Y = 7.1819 - 0.1966*X + 0.0141*X^2$
2	8,982.97	.85	$Y = 4.9685 - 0.0608*x + 0.0012*X^2$<br>$Y = 9.3093 - 0.3283*x + 0.0267*X^2$
3	9,018.32	.80	$Y = 4.1343 - 0.1223*X + 0.0056*X^2$<br>$Y = 6.7179 + 0.08*X - 0.0047*X^2$<br>$Y = 10.6954 - 0.5258*X + 0.0395*X^2$
4	9,082.24	.65	$Y = 4.3141 + 0.3974*X - 0.0322*X^2$<br>$Y = 5.9052 - 0.4869*X + 0.0282*X^2$<br>$Y = 6.9689 + 0.3035*X - 0.0105*X^2$<br>$Y = 11.8108 - 1.0304*X + 0.0731*X^2$

Table A2C.
Four models of the Communication-with-physicians data

Number of groups in the model	Model fit statistics		Equation(s) for the group(s) in the model
	BIC	Classification (P_c)	
1	8,690.05	Not applicable	$Y = 6.2124 + 0.1951*X - 0.0124*X^2$
2	8,645.97	.88	$Y = 4.0252 + 0.0842*X - 0.0075*X^2$<br>$Y = 8.4282 + 0.3049*X - 0.0174*X^2$
3	8,671.51	.72	$Y = 3.4292 + 0.1005*X - 0.008*X^2$<br>$Y = 7.108 + 0.3629*X - 0.0197*X^2$<br>$Y = 8.4057 + 0.1401*X - 0.0108*X^2$
4	8,716.24	.68	$Y = 2.9075 + 0.1054*X - 0.0087*X^2$<br>$Y = 6.2069 - 0.0338*X - 0.0034*X^2$<br>$Y = 7.065 + 0.6366*X - 0.0289*X^2$<br>$Y = 8.7784 + 0.1061*X - 0.0103*X^2$

Appendix 3.

Contingency tables for trajectory-group memberships (DoI: decay of impact)

Table A3A. Anxiety and depression

Anxiety group	Depression group: Not DoI	DoI	Total
Not DoI	180	68	248
DoI	45	163	208
Total	225	231	456

Phi coefficient = 0.508

95% CI: 0.420 to 0.585

Table A3B. Anxiety and communication

Anxiety group	Communication group: Not DoI	DoI	Total
Not DoI	104	144	248
DoI	107	101	208
Total	211	245	456

Phi coefficient = –0.095

95% CI: –0.190 to 0.001

Table A3C. Depression and communication

Depression group	Communication group: Not DoI	DoI	Total
Not DoI	109	116	225
DoI	102	129	231
Total	211	245	456

Phi coefficient = 0.043

95% CI: –0.053 to 0.138
