# Supplementary material for: Trajectories of change after a health-education program in Japan: decay of impact in anxiety, depression, and patient-physician communication
Source: PeerJ. 2019 Jul 15;7:e7229. doi: 10.7717/peerj.7229 (PMC6637923; doi:10.7717/peerj.7229)
Supplement: Supplemental Information 2 — This document has both Japanese and English versions of the four questionnaires: self-efficacy, anxiety, depression, and communication with physicians. [file peerj-07-7229-s002.pdf]

Self-efficacy

【実行できる自信について】

現時点で、あなたが次のことを実行できる自信はどのくらいありますか。

（それぞれあてはまる数字ひとつに○）

1）病気による疲労があっても
やりたいことを実行できる自信は
どのくらいありますか？

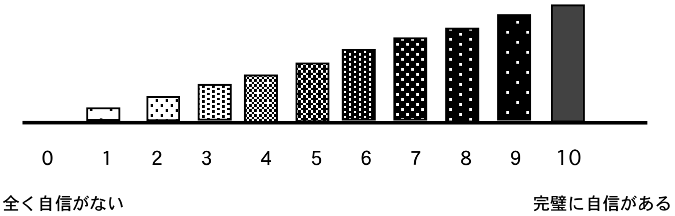

2）病気による体の不快さや痛みがあっても
やりたいことを実行できる自信は
どのくらいありますか？

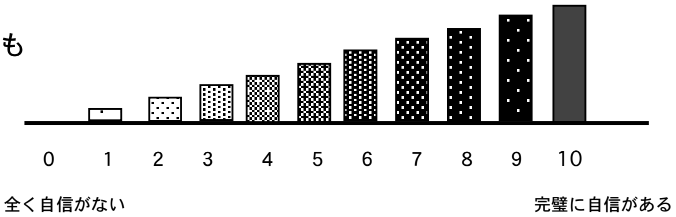

3）病気による精神的苦痛があっても
やりたいことを実行できる自信は
どのくらいありますか？

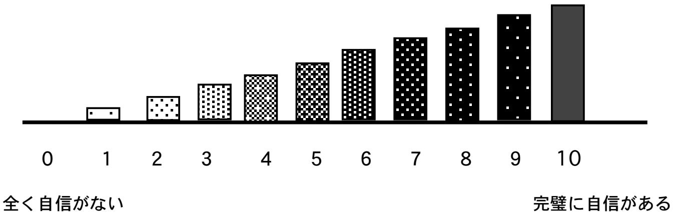

4）その他の症状や健康問題があっても
やりたいことを実行できる自信は
どのくらいありますか？

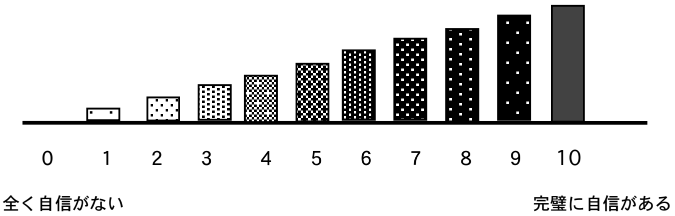

5）医師にかかる回数が減るように
あなた自身の健康管理に必要な
さまざまなことを実行できる自信は
どのくらいありますか？

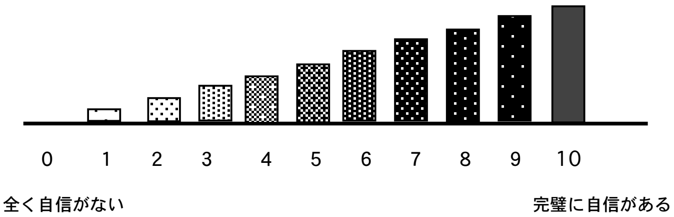

6）病気による日常生活への影響が減るように
服薬以外のことも実行できる自信は
どのくらいありますか？

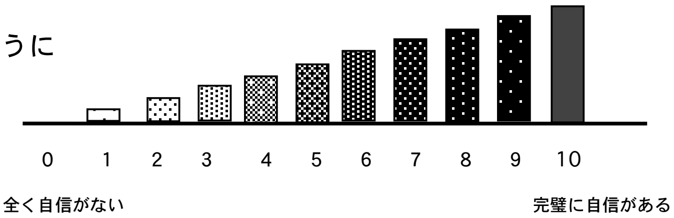

SMRC
Self-Management
Resource Center

Self-Efficacy for Managing Chronic Disease 6-item Scale

We would like to know how confident you are in doing certain activities. For each of the following questions, please choose the number that corresponds to your confidence that you can do the tasks regularly at the present time.

1. How confident do you feel that you can keep the fatigue caused by your disease from interfering with the things you want to do?

   not at all confident | 1 | 2 | 3 | 4 | 5 | 6 | 7 | 8 | 9 | 10 | totally confident

2. How confident do you feel that you can keep the physical discomfort or pain of your disease from interfering with the things you want to do?

   not at all confident | 1 | 2 | 3 | 4 | 5 | 6 | 7 | 8 | 9 | 10 | totally confident

3. How confident do you feel that you can keep the emotional distress caused by your disease from interfering with the things you want to do?

   not at all confident | 1 | 2 | 3 | 4 | 5 | 6 | 7 | 8 | 9 | 10 | totally confident

4. How confident do you feel that you can keep any other symptoms or health problems you have from interfering with the things you want to do?

   not at all confident | 1 | 2 | 3 | 4 | 5 | 6 | 7 | 8 | 9 | 10 | totally confident

5. How confident do you feel that you can the different tasks and activities needed to manage your health condition so as to reduce your need to see a doctor?

   not at all confident | 1 | 2 | 3 | 4 | 5 | 6 | 7 | 8 | 9 | 10 | totally confident

6. How confident do you feel that you can do things other than just taking medication to reduce how much your illness affects your everyday life?

   not at all confident | 1 | 2 | 3 | 4 | 5 | 6 | 7 | 8 | 9 | 10 | totally confident

Scoring

The score for each item is the number circled. If two consecutive numbers are circled, code the lower number (less self-efficacy). If the numbers are not consecutive, do not score the item. The score for the

scale is the mean of the six items. If more than two items are missing, do not score the scale. Higher number indicates higher self-efficacy.

Characteristics

Tested on 605 subjects with chronic disease
.

No. of items	Observed Range	Mean	Standard Deviation	Internal Consistency Reliability	Test-Retest Reliability
6	1-10	5.17	2.22	.91	NA

Source of Psychometric Data

Stanford/Garfield Kaiser Chronic Disease Dissemination Study. Psychometrics reported in: Lorig KR, Sobel, DS, Ritter PL, Laurent, D, Hobbs, M. Effect of a self-management program for patients with chronic disease. *Effective Clinical Practice*, 4, 2001,pp. 256-262.

Comments

This 6-item scale contains items taken from several SE scales developed for the Chronic Disease Self-Management study. We use this scale now, as it is much less burdensome for subjects. It covers several domains that are common across many chronic diseases, symptom control, role function, emotional functioning and communicating with physicians. For internet studies, we add radio buttons below each number. There are 2 ways to format these items. We use the format on this document, the other is shown on the web page. A 4-item version of this scale available in Spanish.

References

Lorig KR, Sobel, DS, Ritter PL, Laurent, D, Hobbs, M. Effect of a self-management program for patients with chronic disease. *Effective Clinical Practice*, 4, 2001,pp. 256-262.

This scale is free to use without permission

Self-Management Resource Center
711 Colorado Avenue
Palo Alto CA 94303
(650) 242-8040
smrc@selfmanagementresource.com
www.selfmanagementresource.com

Anxiety & depression

【心の状態について】

ここ１週間のあなたのご様子についてうかがいます。
あなたはここ１週間どのように感じていますか。（それぞれあてはまる番号 ひとつに○）

	0	1	2	3
１．緊張したり、気持ちが張りつめたりすることが	しょっちゅうあった	たびたびあった	ときどきあった	全くなかった
２．むかし楽しんだことを今でも楽しいと思うことが	全く同じだけあった	かなりあった	少しだけあった	めったになかった
３．なにか恐ろしいことが起ころうとしているという恐怖感を持つことが	しょっちゅうあって非常に気になった	たびたびあるがあまり気にならなかった	少しあるが気にならなかった	全くなかった
４．物事の面白い面を笑ったり、理解したりすることが	いつもと同じだけできた	かなりできた	少しだけできた	全くできなかった
５．心配事が心に浮かぶことが	しょっちゅうあった	たびたびあった	それほど多くはないが、ときどきあった	ごくたまにあった
６．きげんの良いことが	全くなかった	たまにあった	ときどきあった	しょっちゅうあった
７．楽に座って、くつろぐことが	かならずできた	たいていできた	たまにできた	全くできなかった
８．仕事を怠けているように感じることが	ほとんどいつもあった	たびたびあった	ときどきあった	全くなかった
９．不安で落ち着かないような恐怖感を持つことが	全くなかった	ときどきあった	たびたびあった	しょっちゅうあった
１０．自分の顔、髪型、服装に関して	関心がなくなった	以前より気を配っていなかった	以前ほどは気を配っていなかったかもしれない	いつもと同じように気を配っていた
１１．じっとしていられないほど落ち着かないことが	しょっちゅうあった	たびたびあった	少しだけあった	全くなかった
１２．物事を楽しみにして待つことが	いつもと同じだけあった	以前ほどはなかった	以前よりも明らかに少なかった	めったになかった
１３．突然、理由のない恐怖感（ﾊﾟﾆｯｸ）におそわれることが	しょっちゅうあった	たびたびあった	少しだけあった	全くなかった
１４．面白い本や、ラジオまたはテレビ番組を楽しむことが	たびたびできた	ときどきできた	たまにできた	ほとんどめったにできなかった

Hospital Anxiety and Depression Score (HADS)

This questionnaire helps your physician to know how you are feeling. Read every sentence. Place an “X” on the answer that best describes how you have been feeling during the LAST WEEK. You do not have to think too much to answer. In this questionnaire, spontaneous answers are more important

**A**	**I feel tense or ‘wound up’:**	
	Most of the time	3
	A lot of the time	2
	From time to time (occ.)	1
	Not at all	0
**D**	**I still enjoy the things I used to enjoy:**	
	Definitely as much	0
	Not quite as much	1
	Only a little	2
	Hardly at all	3
**A**	**I get a sort of frightened feeling as if something awful is about to happen:**	
	Very definitely and quite badly	3
	Yes, but not too badly	2
	A little, but it doesn’t worry me	1
	Not at all	0
**D**	**I can laugh and see the funny side of things:**	
	As much as I always could	0
	Not quite so much now	1
	Definitely not so much now	2
	Not at all	3
**A**	**Worrying thoughts go through my mind:**	
	A great deal of the time	3
	A lot of the time	2
	From time to time, but not often	1
	Only occasionally	0
**D**	**I feel cheerful:**	
	Not at all	3
	Not often	2
	Sometimes	1
	Most of the time	0
**A**	**I can sit at ease and feel relaxed:**	
	Definitely	0
	Usually	1
	Not often	2
	Not at all	3

**D**	**I feel as if I am slowed down:**	
	Nearly all the time	3
	Very often	2
	Sometimes	1
	Not at all	0
**A**	**I get a sort of frightened feeling like ”butterflies” in the stomach:**	
	Not at all	0
	Occasionally	1
	Quite often	2
	Very often	3
**D**	**I have lost interest in my appearance:**	
	Definitely	3
	I don’t take as much care as I should	2
	I may not take quite as much care	1
	I take just as much care	0
**A**	**I feel restless as I have to be on the move:**	
	Very much indeed	3
	Quite a lot	2
	Not very much	1
	Not at all	0
**D**	**I look forward with enjoyment to things:**	
	As much as I ever did	0
	Rather less than I used to	1
	Definitely less than I used to	2
	Hardly at all	3
**A**	**I get sudden feelings of panic:**	
	Very often indeed	3
	Quite often	2
	Not very often	1
	Not at all	0
**D**	**I can enjoy a good book or radio/TV program:**	
	Often	0
	Sometimes	1
	Not often	2
	Very seldom	3

Communication with physicians

【医療との関わりについて】

１．あなたが医師を受診する際、次のことをどのくらい行いますか。（それぞれあてはまる番号ひとつに○）

	全くしない	たまにする	時々する	よくする	ほとんどいつもする	いつもする
１）医師に質問したいことのリストを用意する	0	1	2	3	4	5
２）治療について知りたいことや理解できていないことを質問する	0	1	2	3	4	5
3）病気にかかわるあなたの個人的な問題について話し合う	0	1	2	3	4	5

When you visit your doctor, how often do you do the following? (Please circle **one** number for each question.)

Never	*Almost never*	*Sometimes*	*Fairly often*	*Very often*	*Always*
0	1	2	3	4	5

1. Prepare a list of questions for your doctor?
2. Ask questions about the things you want to know and the things you don't understand about your treatment?
3. Discuss any personal problems that may be related to your illness?

Copyright 1996 by Sage Publications, Inc
