# Supplementary material for: Trajectories of change after a health-education program in Japan: decay of impact in anxiety, depression, and patient-physician communication
Source: PeerJ. 2019 Jul 15;7:e7229. doi: 10.7717/peerj.7229 (PMC6637923; doi:10.7717/peerj.7229)
Supplement: Supplemental Information 3 — This is the codebook for interpreting the names of the variables in the data file and their meanings. [file peerj-07-7229-s003.pdf]

Column letter	Column name	Meaning
A	GMM_PeerJ_ID	Serial ID numbers, starting at 1
B	Gender	2 for women, 1 for men
C	Age	In years
D	College?	1 indicates having finished college, 0 indicates all others
E	Marital status	1 indicates married and living together with spouse ("partnered" in the Tables), and 0 indicates all others
F	DM	Diabetes, yes or no?
G	CVD	Cardiovascular disease, yes or no?
H	Rheumatoid	Rheumatoid arthritis, yes or no?
I	CTD	Connective tissue disease, yes or no?
J	FMS	Fibromyalgia syndrome, yes or no?
K	Asthma	Asthma, yes or no?
L	Allergic	Allergic disease, yes or no?
M	pulmonaryDx	Pulmonary disease, yes or no?
N	Cancer	Cancer, yes or no?
O	Depression	Depression, yes or no?
P	Parkinson	Parkinson's disease, yes or no?
Q	IBD	Inflammatory bowel disease, yes or no?
R	NumOthers	Number of other diagnoses
S	NumDx	Total number of diagnoses
T	Historyyear	Length of time, in years, since the first diagnosis of any chronic condition
U	SE0m	Self-efficacy at baseline (0 months)
V	SE3m	Self-efficacy 3 months after baseline
W	SE6m	Self-efficacy 6 months after baseline
X	SE12m	Self-efficacy 12 months after baseline
Y	Anxiety0m	Anxiety at baseline (0 months)
Z	Anxiety3m	Anxiety 3 months after baseline
AA	Anxiety6m	Anxiety 6 months after baseline
AB	Anxiety12m	Anxiety 12 months after baseline
AC	Depression0m	Depression at baseline (0 months)
AD	Depression3m	Depression 3 months after baseline
AE	Depression6m	Depression 6 months after baseline
AF	Depression12m	Depression 12 months after baseline
AG	ComMD0m	Communication with physicians at baseline (0 months)
AH	ComMD3m	Communication with physicians 3 months after baseline
AI	ComMD6m	Communication with physicians 6 months after baseline
AJ	ComMD12m	Communication with physicians 12 months after baseline
